# Pharmacokinetics and safety evaluation of intravenously administered *Pseudomonas* phage PA_LZ7 in a mouse model

**DOI:** 10.1128/spectrum.01882-23

**Published:** 2023-11-28

**Authors:** Si-yun Wang, Xin Tan, Zi-qiang Liu, Hui Ma, Tian-bin Liu, Yong-qing Yang, Yong Ying, Ru-yue Gao, Dai-zhou Zhang, Ying-fei Ma, Kai Chen, Lin Lin, Zhi-huan Jiang, Jia-lin Yu

**Affiliations:** 1 Department of Neonatology, Children’s Hospital of Chongqing Medical University, National Clinical Research Center for Child Health and Disorders, Ministry of Education Key Laboratory of Child Development and Disorders, Chongqing Key Laboratory of Child Infection and Immunity, Chongqing Key Laboratory of Pediatrics, Chongqing, China; 2 Shenzhen Key Laboratory of Synthetic Genomics, Guangdong Provincial Key Laboratory of Synthetic Genomics, CAS Key Laboratory of Quantitative Engineering Biology, Shenzhen Institute of Synthetic Biology, Shenzhen Institutes of Advanced Technology, Chinese Academy of Sciences, Shenzhen, China; 3 New Drug Evaluation Center of Shandong Academy of Pharmaceutical Sciences, Shandong Academy of Pharmaceutical Sciences, Ji'nan, China; 4 Shandong Innovation Center of Engineered Bacteriophage Therapeutics, Ji'nan, China; 5 Department of Neonatology, Southern University of Science and Technology Hospital, Shenzhen, China; Institut Pasteur, Paris, France

**Keywords:** phage therapy, safety, antibiotic resistance, pharmacokinetics

## Abstract

Phage therapy has become one of the most promising alternatives to antibiotics as the surge of multi-drug-resistant (MDR) bacteria severely threatens global public health. The lack of a thorough understanding of pharmacokinetics (PK) and the safety study of phage has limited its clinical application. In this study, we aimed to evaluate the PK and safety of a lytic phage PA_LZ7 that infects MDR *Pseudomonas aeruginosa* using intravenous (IV) route in an uninfected immune-competent mice model. The active phage of PA_LZ7 exhibited an exponential decay in plasma at 2*10^8^ (low dosage), 2*10^10^ [medium dosage (MD)], and 2*10^11^ PFU/kg [high dosage (HD)] after IV administration, and the phage titer dropped to ~4 log_10_ PFU/mL over 24 h for all dosages. Phages primarily accumulated in the spleen at 1 h post-administration and gradually decreased in all dosages. The active phage titer in other organs decreased globally within 72 h as well. Plasma cytokines IL-6, IL-10, KC/GRO, and tumor necrosis factor-alpha sharply increased 1 h post-administration in mice of MD and HD groups and dropped to a normal range within 24 h. No obvious toxic effect was observed, except for the increased spleen weight and relative spleen weight in the MD and HD group and the slight lymphocytosis in the white pulp of the spleen in the HD group. In conclusion, PA_LZ7 possesses good PK properties and shows no obvious toxicity; it could be considered as a potent therapeutic candidate for the treatment of MDR *P. aeruginosa* infections in future phage therapy.

**IMPORTANCE:**

Phage therapy is gaining traction as an alternative to antibiotics due to the rise of multi-drug-resistant (MDR) bacteria. This study assessed the pharmacokinetics and safety of PA_LZ7, a phage targeting MDR *Pseudomonas aeruginosa*, in mice. After intravenous administration, the phage showed an exponential decay in plasma and its concentration dropped significantly within 24 h for all dosage groups. Although there was a temporary increase in certain plasma cytokines and spleen weight at higher dosages, no significant toxicity was observed. Therefore, PA_LZ7 shows potential as an effective and safe candidate for future phage therapy against MDR *P. aeruginosa* infections.

## INTRODUCTION

*Pseudomonas aeruginosa* is one of the main pathogens of hospital-acquired infections (1). *P. aeruginosa* can cause burn site, surgical site, respiratory, and urinary tract infections (2, 3). In recent years, *P. aeruginosa* has shown resistance to a variety of antibiotics (4). Treatment of infections caused by *P. aeruginosa* has become increasingly difficult. WHO publishes a list of bacteria for which new antibiotics are urgently needed. Notably, carbapenem-resistant *P. aeruginosa* is listed as a critically important drug-resistant bacteria (5). Facing the increasing threat of multi-drug-resistant (MDR) *P. aeruginosa*, finding new antimicrobial agents to serve as antibiotic alternatives or supplementary is urgently needed.

Bacteriophages (phages), a kind of natural predator of bacteria, are regarded as a potential antimicrobial treatment and is regarded as one of the most promising alternatives for antibiotic crisis (6 – 9). Both animal models and clinical studies have not reported severe adverse effects of phage therapy (9, 10). However, its widespread clinical implementation has been approached with caution due to unresolved safety considerations (9, 10). This is because phage particles, which are composed of proteins and nucleic acids (DNA or RNA), are much larger than antibiotic molecules and can cause innate and adaptive immune responses in the mammalian body (11, 12). Furthermore, the poor understanding of the pharmacokinetics (PK) of phages *in vivo* also limits their use in clinical. Phages, given their ability to self-replicate, display a unique three-part dynamic interaction with host bacteria and the animal immune system, differentiating them from traditional antimicrobials (11, 13). While the nuanced dynamics between phages and bacteria have been delineated in mathematical models and observed in *in vitro* systems (14, 15), achieving a satisfied phage PK result *in vivo* in the presence of host bacteria remains challenging due to this intricate tripartite interaction. Notably, assessing phage PK in uninfected animals provides essential insights into their behavior devoid of bacterial interference. This foundational understanding aids in predicting phage responses in infected scenarios, steering tissue distribution, clearance, stability, dosage, and potential immune interactions.

Thus, in this study, we aimed to evaluate the PK and safety of a lytic *Pseudomonas* phage PA_LZ7 (GenBank ID: ON759747) that infects MDR *P. aeruginosa* using intravenous (IV) route in an uninfected immune-competent mice model.

## RESULTS

### Pharmacokinetics of intravenously administered PA_LZ7

**Fig 1:**
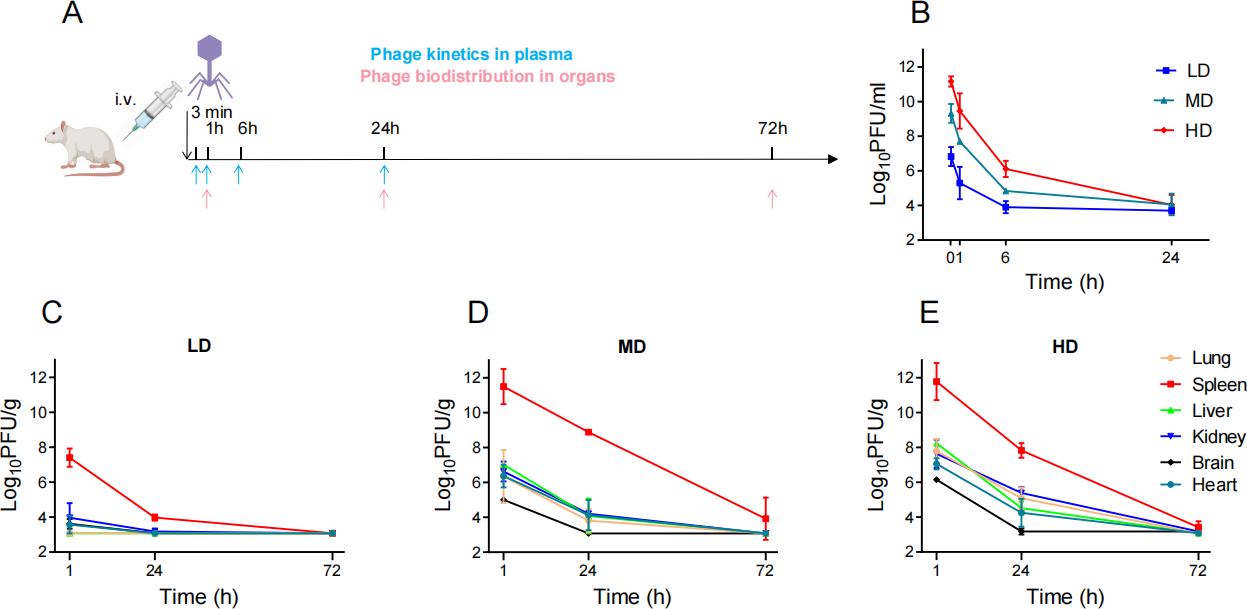
Pharmacokinetics and biodistribution of PA_LZ7 following intravenous administration in ICR mice. (**A**) Schematic representation of the experimental design. Phage kinetics in plasma and phage biodistribution in organs were performed following PA_LZ7 IV administration in mice. (**B**) Kinetics of active phages PA_LZ7 in plasma following IV administration at a dosage of 2*10^8^ (LD), 2*10^10^ (MD), and 2*10^11^ PFU /kg (HD) in healthy ICR mice. Phage titer was obtained by plaque assay, phage titer is expressed as plaque-forming units per milliliter in plasma, and the lower limit of quantification (LLQ) is 5,000 PFU/mL. Biodistribution of active phages in organs following single IV administration in healthy mice from LD (**C**), MD (**D**), and HD (**E**) groups. Active phage titer is expressed as plaque-forming units per gram of each organ; the LLQ is 1,200 PFU/g; phage titer was determined by plaque assay, and each symbol represents the means with standard deviation (SD) (*n* = 3).

Phage PA_LZ7 demonstrated a robust ability to inhibit the growth of *P. aeruginosa* strain PAO1 during a 7-hour co-incubation *in vitro* (Fig. S1). We examined the PK of PA_LZ7 administered through IV route in an ICR mouse model (Fig. 1A). The dosages of phage administration for the study were based on previous pharmacological testing, which used IV administration with 5*10^8^ to 5*10^10^ PFU/kg/day in different animal models to demonstrate the efficacy of phage therapy (16 – 18). Indeed, phage was typically IV administered with 10^9^ PFU (about 2*10^7^ PFU/kg) per dose in clinical settings (6, 19, 20). To examine the safety of phages in animals, we used three dosages of 2*10^8^ [low dosage (LD)], 2*10^10^ [medium dosage (MD)], and 2*10^11^ PFU/kg [high dosage (HD)] in this study.

Following IV administration of phages, Fig. 1B showed the concentration-time profiles of active phages. Overall, the plasma active phage titer gradually decreased over time regardless of the dosage that was given. At 3 min after administration, the phage titer was 6.28 ± 0.55, 9.32 ± 0.55, and 11.17 ± 0.30 log_10_ PFU/mL in the LD, MD, and HD groups, respectively. At 24 h, the titer dropped to the lower limit of quantification (LLQ), ~4 log_10_ PFU/mL, in all three groups (Fig. 1B). The elimination of active phage could be adequately described using a simple exponential decay model, with the weighted *R^2^* being 0.99, 1, and 1 for the LD, MD, and HD, respectively (16). The elimination rate constant *k* was estimated to be 3.092 h^−1^ [95% confidence interval (CI), 2.511 to 4.318) for the LD group, 4.493 h^−1^ (95% CI, 4.235 to 4.833) for the MD group, and 3.258 h^−1^ (95% CI, 3.069 to 3.483) for the HD group, which are equivalent to half-lives of 0.224 (95% CI, 0.1605 to 0.2761) and 0.154 (95% CI, 0.1434 to 0.1637), and 0.213 (95% CI, 0.199 to 0.2258) h, respectively.

Biodistribution of active phages in organs following single IV administration in healthy mice was also evaluated. At 1, 24, or 72 h, mice were sacrificed, and tissues were harvested for phage PA_LZ7 quantification. Phages primarily accumulated in the spleen at 1 h post administration and gradually decreased in all dosages (Fig. 1C through E). The active phage titer in other organs decreased globally within 72 h as well. For example, in HD group, at 1 h post administration, the spleen had substantially higher active phage titer (11.78 ± 1.06 log_10_ PFU/g, *P* < 0.0001) than the liver (8.24 ± 0.09 log_10_ PFU/g), the lung (7.79 ± 0.70 log_10_ PFU/g), kidney (7.63 ± 0.82 log_10_ PFU/g), brain (6.14 ± 0.06 log_10_ PFU/g), and heart (7.05 ± 0.32 log_10_ PFU/g) (Fig. 1E); at 72 h, the active phage titer in all organs dropped to 3–4 log_10_ PFU/g (Fig. 1E).

### Safety study of intravenously administered PA_LZ7

**Fig 2:**
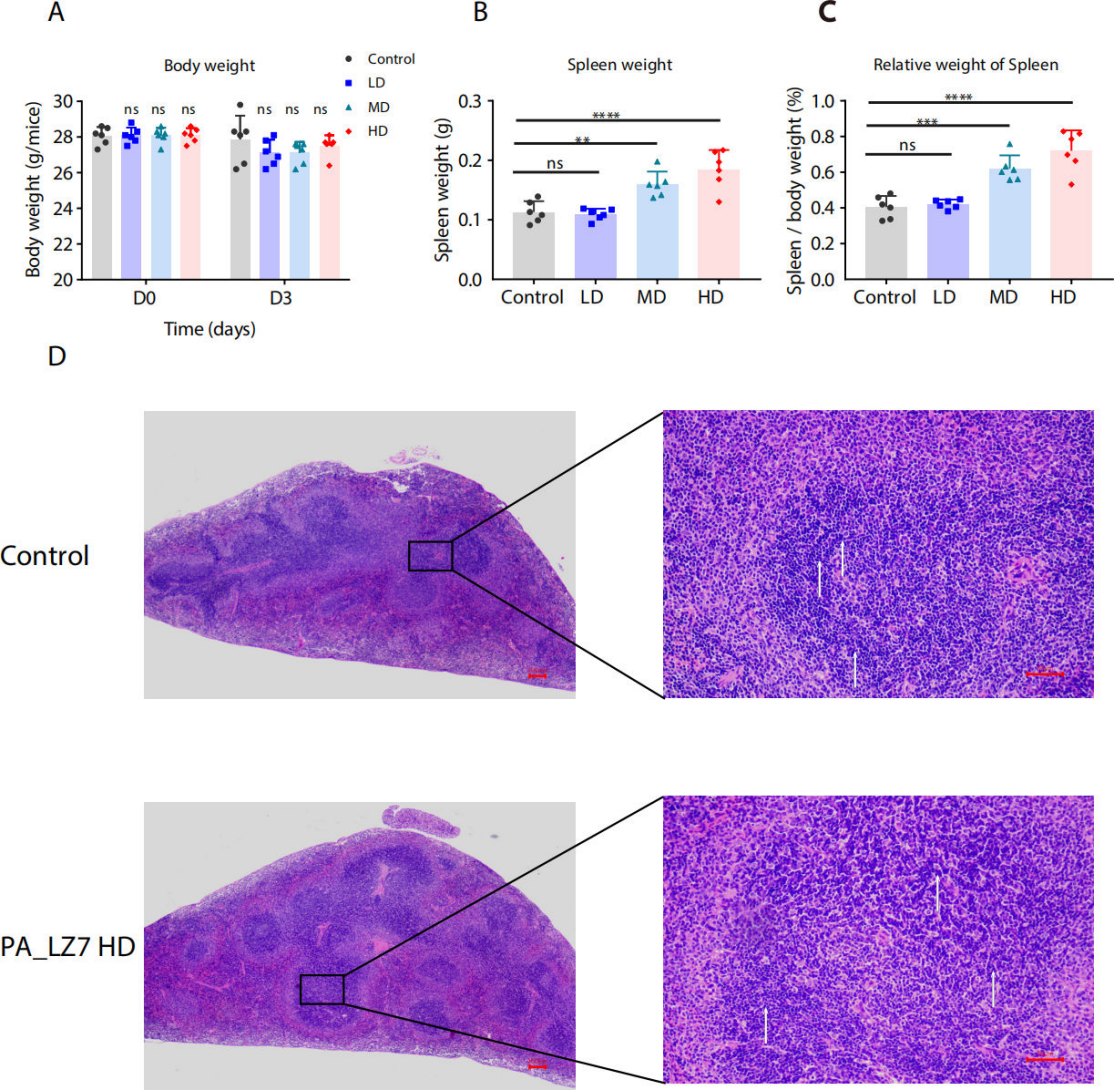
Effect of PA_LZ7 IV administration on the body weight, spleen weight, the relative weight of spleen, and splenic histology of ICR mice exposed to two doses of PA_LZ7. (**A**) The body weight of the animal was measured on D0 and D3. (**B**)Spleen weight was measured on the day of sacrifice (**D4**). (**C**) Relative weight of spleen was calculated as (spleen weight (g)/body weight of animal on D3 (g)) × 100. Data are presented as means with SD. The comparison was performed between PA_LZ7-treated and control groups (*n* = 6; **P < 0.05; ***P < 0.001; ns, no significance). (**D**) Representative hematoxylin and eosin (HE)-stained section of the spleen. More lymphocytes, characterized by their dark blue, round nuclei (indicated by white arrows in the right panel), are observed in the white pulp of the phage-treated group. In the left panel, the scale bar represents 50 µm, while in the right panel, it represents 100 µm.

To determine the safety of PA_LZ7, female ICR mice were administered PA_LZ7 intravenously with two doses at dosages 0 (vehicle control), LD, MD, or HD. During the 4-day experimental period, mortality and body mass were monitored, and a necropsy was undertaken at the end of the experimental period. No deaths or clinical signs were observed. In addition, there were no significant differences in the body mass of the PA_LZ7-treated groups compared with the vehicle control group (Fig. 2A ; Tables 1 and 2). No abnormalities were found during necropsy, except the spleen weight increased significantly in the MD (*P* < 0.01) and HD (*P* < 0.0001) groups (Fig. 2B), with increasing relative spleen weight (RSW) (*P* < 0.001) in both groups (Fig. 2C). Furthermore, PA_LZ7 had no significant hematologic, serum biochemical, or coagulation effects on the physiological parameters (Tables 3 to 5).

**TABLE 1:** Effect of body or organ weight for mice exposed to two doses of PA_LZ7^*a*^

Item	Control (g)	LD (g)	MD (g)	HD (g)
Body	28.0 ± 1.5	25.9 ± 1.0	25.7 ± 0.7	25.6 ± 0.8
Heart	0.133 ± 0.015	0.118 ± 0.005	0.125 ± 0.009	0.126 ± 0.011
Liver	1.400 ± 0.259	1.052 ± 0.106^*c*^	1.089 ± 0.070^*c*^	1.120 ± 0.083^*b*^
Spleen	0.113 ± 0.018	0.109 ± 0.010	0.160 ± 0.022^*c*^	0.185 ± 0.033^*d*^
Kidney	0.358 ± 0.057	0.335 ± 0.024	0.336 ± 0.031	0.323 ± 0.023^*b*^
Thymus	0.070 ± 0.010	0.068 ± 0.012	0.065 ± 0.019	0.066 ± 0.013
Brain	0.467 ± 0.039	0.468 ± 0.025	0.469 ± 0.024	0.463 ± 0.015

^*a*^ Body weight of animal was measured on D3. Data are presented as the means with SD (*n* = 6).

^*b*^ *P* < 0.05.

^*c*^ *P* < 0.01.

^*d*^ *P* < 0.0001.

**TABLE 2:** Effect of relative organ weight for mice exposed to two doses of PA_LZ7^*a*^

Item	Control (%)	LD (%)	MD (%)	HD (%)
Heart	0.474 ± 0.050	0.456 ± 0.020	0.486 ± 0.035	0.490 ± 0.036
Liver	4.981 ± 0.746	4.062 ± 0.304^*c*^	4.245 ± 0.216^*b*^	4.376 ± 0.247
Spleen	0.405 ± 0.062	0.421 ± 0.025	0.621 ± 0.074^*d*^	0.721 ± 0.114^*e*^
Kidney	1.377 ± 0.190	1.296 ± 0.076	1.310 ± 0.121	1.260 ± 0.066
Thymus	2.492 ± 0.303	2.632 ± 0.375	2.522 ± 0.734	2.586 ± 0.514
Brain	1.672 ± 0.182	1.817 ± 0.163	1.829 ± 0.086	1.811 ± 0.081

^*a*^ Relative organ weight was calculated as (organ weight (g)/body weight of animal on D3 (g)) × 100. Data are presented as the means with SD (*n* = 6).

^*b*^ *P* < 0.05.

^*c*^ *P* < 0.01.

^*d*^ *P* < 0.001.

^*e*^ *P* < 0.0001.

**TABLE 3:** Effect of hematologic values for mice exposed to two doses of PA_LZ7^*a*^

Parameter^*c*^	Control	LD	MD	HD
WBC (10^9^ /L)	2.46 ± 0.28	2.38 ± 0.65	2.85 ± 0.80	4.11 ± 0.44^*b*^
RBC (10^12^ /L)	7.90 ± 0.20	8.42 ± 0.54	8.31 ± 0.38	8.72 ± 0.40
HGB (g/L)	133 ± 2	130 ± 1	134 ± 4	138 ± 7
PLT (10^9^ /L)	1436 ± 93	1441 ± 119	1666 ± 155	1316 ± 172
HCT (%)	40.80 ± 0.28	40.53 ± 1.03	40.47 ± 0.81	41.87 ± 1.78
MCV (fL)	51.65 ± 0.92	48.23 ± 1.84	48.80 ± 2.26	48.03 ± 0.25
MCH (pg)	16.80 ± 0.14	15.53 ± 0.86	16.13 ± 0.95	15.83 ± 0.09
MCHC (g/L）	325.00 ± 2.83	321.67 ± 4.99	330.33 ± 4.64	329.67 ± 2.49
NEU% (%)	15.10 ± 15.56	15.33 ± 9.29	22.33 ± 14.46	13.53 ± 0.09
LYM% (%)	64.8 ± 43.42	71.93 ± 3.37	65.73 ± 25.94	72.93 ± 10.36
MON% (%)	19.70 ± 27.86	11.87 ± 10.96	9.87 ± 10.61	12.07 ± 2.09
EOS% (%)	0.4 ± 0	0.70 ± 0.62	1.83 ± 1.10	1.03 ± 0.74
BAS% (%)	0 ± 0	0.17 ± 0.24	0.23 ± 0.17	0.43 ± 0.17
RET% (%)	4.40 ± 1.32	5.55 ± 0.85	4.79 ± 1.33	5.10 ± 1.31
NEU (10^9^ /L)	0.35 ± 0.34	0.31 ± 0.17	0.69 ± 0.44	0.57 ± 0.42
LYM (10^9^ /L)	1.66 ± 1.25	1.70 ± 0.41	1.81 ± 0.76	2.99 ± 0.49
MON (10^9^ /L)	0.45 ± 0.63	0.35 ± 0.38	0.29 ± 0.29	0.49 ± 0.06
EOS (10^9^ /L)	0.01 ± 0	0.02 ± 0.02	0.06 ± 0.03	0.04 ± 0.02
BAS (10^9^ /L)	0 ± 0	0.03 ± 0.05	0.007 ± 0.005	0.017 ± 0.005^*b*^
RET (10^12^ /L)	0.35 ± 0.10	0.47 ± 0.09	0.40 ± 0.13	0.45 ± 0.13

^*a*^ Blood was collected on sacrifice day (D4). Data are presented as means with SD (*n* = 3).

^*b*^ *P* < 0.05.

^*c*^ WBC: white blood cell; RBC: red blood cell; HGB: hemoglobin; PLT: platelet; HCT: hematocrit; MCV: mean corpuscular volume; MCH: mean corpuscular hemoglobin; MCHC: mean corpuscular hemoglobin concentration; NEU: neutrophil; LYM: lymphocyte; MON: monocyte; EOS: eosinophil; BAS: basophil; RET: reticulocyte.

**TABLE 4:** Effect of serum biochemical parameters for mice exposed to two doses of PA_LZ7^*a*^

Parameter^*e*^	Control	LD	MD	HD
ALT (U/L)	68.2 ± 26.7	50.9 ± 16.0	36.8 ± 2.5	34.8 ± 1.5
AST (U/L)	115.1 ± 31.2	110.9 ± 42.3	104.0 ± 17.6	75.9 ± 24.8
TP (g/L)	48.1 ± 1.1	47.0 ± 2.0	47.6 ± 1.7	45.5 ± 0.6
ALB (g/L)	27.2 ± 0.4	27.0 ± 0.9	25.8 ± 0.9	25.0 ± 0.6
ALP (U/L)	116 ± 16	94 ± 4	67 ± 6^*c*^	64 ± 4^*c*^
GLU (mmoL/L)	17.19 ± 4.29	16.60 ± 2.40	10.20 ± 0.41	11.41 ± 0.09
Urea (mmoL/L)	5.21 ± 0.42	8.13±0.64^*d*^	4.51 ± 0.20	6.82 ± 0.05^*b*^
CREA (μmoL/L)	7.7 ± 1.4	8.1 ± 0.8	8.2 ± 1.6	10.8 ± 1.6
TC (mmoL/L)	3.21 ± 0.50	2.81 ± 0.20	2.64 ± 0.32	2.64 ± 0.03
TG (mmoL/L)	1.80 ± 0.20	1.72 ± 0.14	1.40 ± 0.46	1.37 ± 0.33
CK (U/L)	519 ± 191	651 ± 35	837 ± 135	740 ± 35
K^+^ (mmoL/L)	4.8 ± 0.2	5.9 ± 0.5	5.9 ± 0.4	5.9 ± 0.2
Na^+^ (mmoL/L)	146 ± 0	144 ± 0	145 ± 0	146 ± 2
Cl^-^ (mmoL/L)	113 ± 0	113 ± 0	114 ± 2	113 ± 1
GLOB (g/L)	20.9 ± 0.7	20 ± 1.1	21.8 ± 1.0	20.5 ± 0.1
A/G	1.30 ± 0.03	1.36 ± 0.03	1.18 ± 0.04^*b*^	1.22 ± 0.03

^*a*^ Blood was collected on sacrifice day (D4). Data are presented as means with SD (*n* = 3).

^*b*^ *P* < 0.05.

^*c*^ *P* < 0.01.

^*d*^ *P* < 0.001.

^*e*^ ALT: alanine aminotransferase; AST: aspartate aminotransaminase; TP: total protein; ALB: albumin; ALP: alkaline phosphatase; GLU: glucose; CREA: creatinine; TC: total cholesterol; TG: triglyceride; CK: creatine kinase; GLOB: globulin; A/G: albumin/globulin.

**TABLE 5:** Effect of blood coagulation parameters for mice exposed to two doses of PA_LZ7^*a*^

Parameter^*b*^	Control	LD	MD	HD
PT (s)	10.00 ± 0.20	10.10 ± 0.16	10.20 ± 0.20	9.93 ± 0.24
APTT (s)	14.00 ± 0.40	12.30 ± 0.57	14.05 ± 0.95	14.87 ± 1.93
FIB (g/L)	1.83 ± 0.15	1.66 ± 0.08	2.12 ± 0.09	2.40 ± 0.12

^*a*^ Blood was collected on sacrifice day (D4). Data are presented as means with SD (*n* = 3).

^*b*^ PT: prothrombin time; APTT: activated prothrombin time; FIB: fibrinogen.

**Fig 3:**
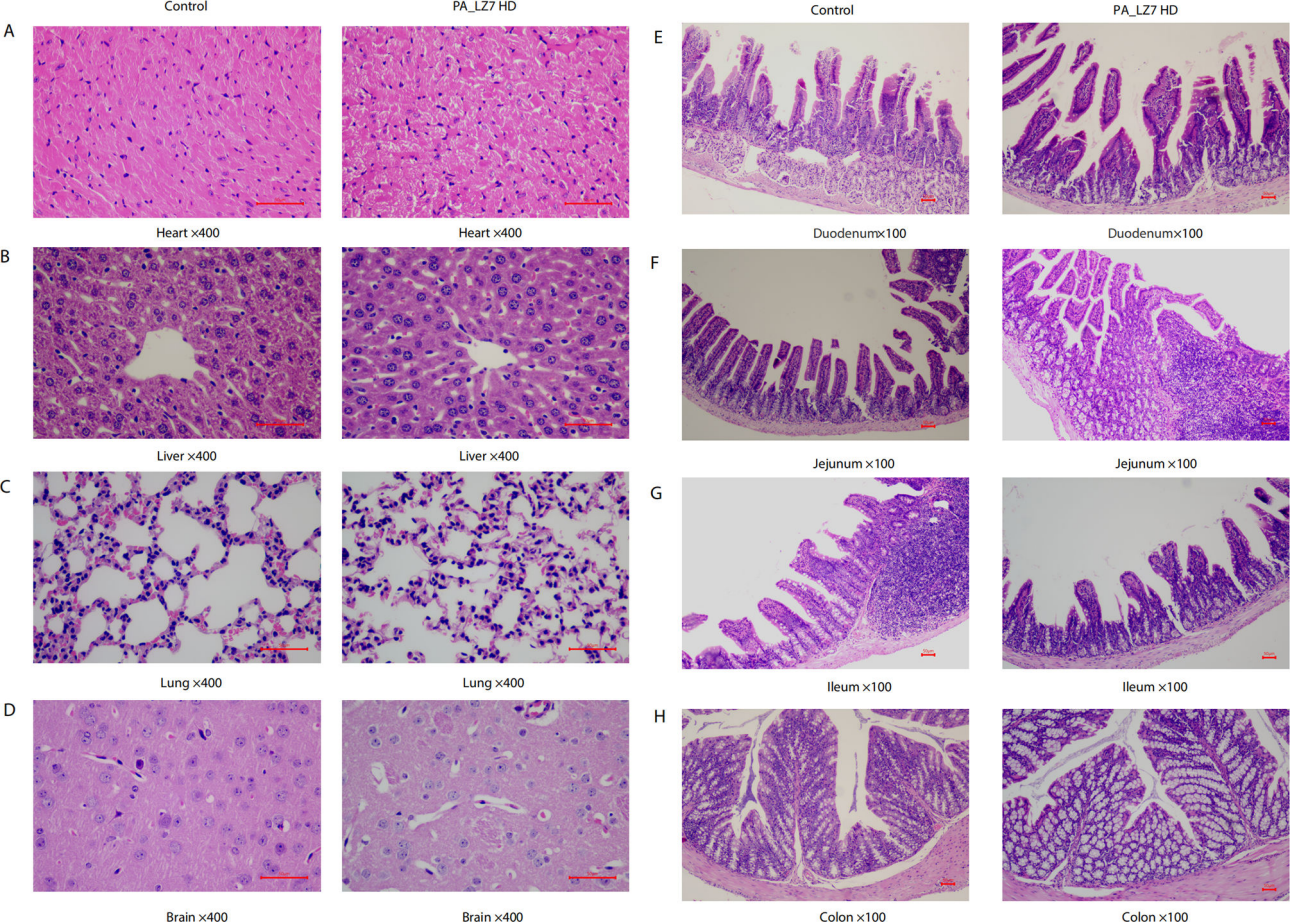
Representative HE-stained section of the normal structure of organs in animals with PBS or PA_LZ7 with HD: heart (**A**), liver (**B**), lung (**C**), brain (**D**), duodenum (**E**), ileum (**F**), jejunum (**G), **and colon (**H**). No significant architectural differences, disparities in cytomorphology, spatial organization, or staining affinities were observed in these tissues with or without phage treatment. The magnifications are indicated in the figure. The scale bar represents 50 µm.

A slight lymphocytosis was observed in the white pulp of the spleen from the HD group (three out of six mice) (Fig. 2D). No clinically significant histopathologic findings were noted in any of the brain, heart, lung, kidney, liver, or gastrointestinal tract samples (Fig. 3).

**Fig 4:**
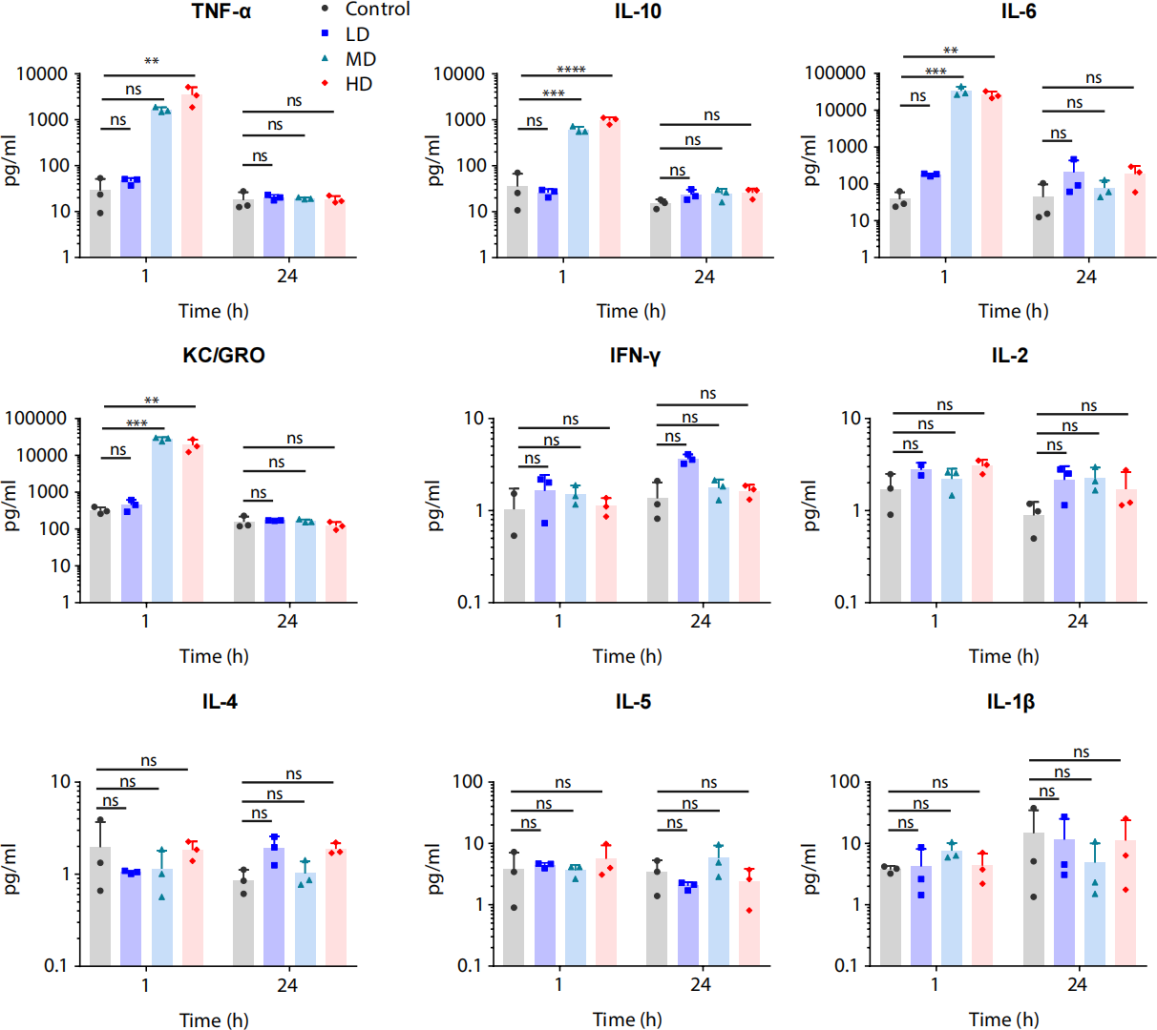
Cytokine profile in plasma of ICR mice following IV phage administration. Cytokine concentrations in plasma for ICR mice were measured at 1 and 24 h with phage PA_LZ7 following a single dose. Data are presented as means with SD. Comparisons performed exclusively within the same time point (*n* = 3; **P < 0.01; ***P < 0.001; ****P < 0.0001; ns, no significance).

To further investigate the potential side effect triggered by PA_LZ7, we measured cytokine concentrations in plasma at 1 and 24 h following phage administration (Fig. 4). The increased cytokines were mainly observed in the MD and HD group mice 1 h post administration, including IL-6, IL-10, tumor necrosis factor-alpha (TNF-α), and keratinocyte chemoattractant/growth-regulated oncogene (KC/GRO) (Fig. 4) and dropped to a normal range within 24 h. For example, we observed that at 1 h post administration, the concentration of IL-6 significantly increased in the MD (nearly 900-fold higher, *P* < 0.001) and HD (about 700-fold higher, *P* < 0.01) group compared to the control group (Fig. 4). The concentrations of IL-10, TNF-α, and KC/GRO responded similarly, but the effect was mild (Fig. 4). In comparison, interferon gamma, IL-1β, IL-2, IL-4, and IL-5 showed no significant variations (Fig. 4).

## DISCUSSION

Phage therapy is considered as one of the promising alternatives for the increasing threat of MDR bacteria (21). However, there are still some obstacles restricting its application, including a lack of PK, systemic safety studies and strict regulation by the Food and Drug Administration. PK and safety studies are the fundamental parameters for obtaining regulatory approval. In this study, we used ICR mouse as a model animal to investigate the PK of a *Pseudomonas* phage PA_LZ7. The active phage of PA_LZ7 exhibited an exponential decay in plasma at LD, MD, and HD after IV administration (Fig. 1B), and the phage titer dropped to ~4 log_10_ PFU/mL over 24 h for all dosages. Further tissue distribution studies have found that PA_LZ7 is mainly accumulated in the spleen 1 h post administration (Fig. 1C through E). After 24 h of administration, compared with other tissues such as the liver, kidney, and lung, the active phage titer in the spleen was still at a higher level, which was consistent with what was reported in the literature (16). The preferential distribution of phage PA_LZ7 to the spleen compared to other organs can be elucidated through the intricate functions of the mononuclear phagocytic system (MPS). The spleen and liver, being a primary organ within the MPS, serve as a nexus for the clearance of foreign entities, including phage particles. It has been well documented that phage clearance in the host is mostly attributable to the phagocytosis of phage particles in the MPS, especially the liver and spleen (11, 22 – 24). Moreover, phages degrade rapidly in the liver, and intact phage particles accumulate in the spleen, where they decay at a slower rate (11, 22 – 24). This differential degradation rate can account for the heightened distribution of phage PA_LZ7 to the spleen compared to the liver.

Importantly, phages, frequently described as “living antibiotics” due to their self-replicating nature, maintain a bidirectional relationship with bacteria. This relationship is influenced by variable phage and bacterial loads. Typically, post infection and replication, the new phage progeny are released to initiate another cycle, enhancing the phage load and amplifying bacterial eradication. However, a reduction in bacterial population can inhibit phage replication, leading to a decreased phage titer and lessened bacterial eradication, which might result in an uptick in bacterial numbers. This dynamic profoundly impacts phage PK and PD. The intertwined interactions between phages and bacteria have been represented in mathematical models and observed in *in vitro* systems (14, 15). Therefore, the reciprocal interactions between phage and bacteria must be taken into account for the study of PK. In the future, more PK studies in animal models infected with the target bacteria are warranted.

Plasma cytokines IL-6, IL-10, KC/GRO, and TNF-α sharply increased 1 h post administration in mice of MD and HD groups (Fig. 4). These cytokines are important indicators of inflammation (25, 26). For example, IL-6 can regulate the growth and differentiation of various cells, regulate immune response and hematopoietic function, and play an important role in the body’s anti-infection immune response (27); TNF-α is secreted by activated macrophages and other cells, which plays an important role for host fight against infection (28). Our results suggest that phage PA_LZ7 preparation can trigger pro-inflammatory reactions after entering the body. Indeed, researchers have shown that several phages can stimulate the immune cells *in situ* and trigger both inflammatory and anti-inflammatory responses (25, 29 – 31).

In the MD and HD groups, we observed the increased spleen weight and RSW (Fig. 2B and C) and the slight lymphocytosis in the white pulp of the spleen in the HD group (Fig. 2D). The RSW is one of the important indicators reflecting the body’s immune function (32, 33). The increased RSW in mice in the MD and HD groups may be due to the immune response of the mice to the phage preparation, resulting in compensatory hyperplasia of the spleen. The mice in this study were sacrificed on the fourth day after the first administration; the RSW can recover to normal range after a recovery period. Despite these observations, our study did not detect any other evident toxic effects (Tables 1–5). Both the white blood cell count and other hematological parameters consistently remained within the normal range. Likewise, biochemical markers indicative of liver, heart, and renal functions were unaltered. These findings imply that, although phages might trigger a temporary inflammatory response, they do not inflict harm on the liver, heart, kidneys, or other organs.

In this study, we purified the phages using an approach that can efficiently remove leftover DNA, RNA, endotoxins, and other bacterial impurities released during the lysis of the bacterial cell (34). Mice received 10, 1,000, and 10,000 EU/kg of endotoxin for LD, MD, and HD groups, respectively, which is higher than the endotoxin concentration allowable limit for preclinical study (<5 EU/kg/h) (35). Although the rodents were far more resistant to endotoxin than human (36, 37), we could not rule out the pro-inflammatory effect of endotoxin for our phage preparation. The phage preparation with lower endotoxin level is warranted in future studies.

Concerns regarding the phage’s safety are raised both by the phage’s immunogenicity and the presence of hazardous leftovers like endotoxin in the phage preparation (25, 29 – 31, 34). It is necessary to optimize the preparation process and quality control indicators. Due to the growing threat of multi-drug-resistant bacterial strains, there is rising interest in phage therapy as a potential solution from academic, clinical, and governmental sectors. Governments are investing in phage therapy research and application, while academics delve into its mechanisms and efficacy. Clinicians are considering it as both a primary and complementary treatment against tough bacterial infections, highlighting its importance in the fight against bacterial super-resistance (38). We believe that with the improvement and standardization of phage preparation research and development and the continuous accumulation of animal and clinical study experience data, phage therapy will become an important weapon against superbugs in the future.

In conclusion, PK of a *Pseudomonas* phage PA_LZ7 *in vivo* was assessed. Half-life and kinetics of the phage in blood and biodistribution in lungs, liver, kidneys, spleen, brain, and heart of mouse model were determined at different dosages and different time intervals via IV routes of administration. Although the relatively high dosages of PA_LZ7 evoke a transient inflammation in plasma, no other obvious phage-related toxic reactions were observed. Therefore, we suggested that PA_LZ7 could be considered as a potent therapeutic candidate for the treatment of MDR *P. aeruginosa* infection. However, more work is necessary to better understand the interaction of body versus PA_LZ7 using lower endotoxin level phage preparation.

## MATERIALS AND METHODS

### Bacterial strains, phages, and media

*P. aeruginosa* reference strain PAO1 and phage PA_LZ7 was used in this work. PA_LZ7 is a lytic *Pseudomonas* phage and belongs to Pbunavirus genus. It (referred as PA39) has been applied in animal and clinical studies and has proven to be highly effective against clinical MDR *P. aeruginosa* infections (39, 40). We cultivated PA_LZ7 with a *P. aeruginosa* reference strain PAO1.

Phage and its host were incubated at 37℃, 220 rpm, using Luria-Bertani (LB) broth without sugar medium (HuanKai Microbial). Soft agar was composed of LB and 0.75% (wt/vol）bacteriological agar powder (HuanKai Microbial), and LB agar plate was solidified with 1.5% (wt/vol) bacteriological agar powder. Phage lysates were cultivated by liquid culture method according to previous literature (41). Bacterial debris in phage lysate was removed by centrifugation at 8,000 rpm for 5 min and then filtrated through a 0.22-µm PES filter (Millipore).

### Phage cultivation, concentration, and purification

A phage lysate with high titer was prepared before the phage purification process. Ten-milliliter bacteria were added to 2-L LB medium and incubated for 2 h at 37°C while shaking at 220 rpm. Added about 1*10^11^ PFU phage into its bacteria host when the bacteria grew to logarithmic phase (OD_600_ = 0.5) and incubated overnight in the same condition. The lysate was centrifugated twice at 8,000 *g* for 15 min for reducing bacterial debris and other contaminants. The supernatant was filtrated by capsule type filter to remove bacteria. Then, the cross-flow filtration with 100-kDa (Satorius) membrane was assembled to concentrate the lysate from 2 L to 50 mL and to filtrate out part of endotoxin. The titer of the lysate was evaluated after each step.

The concentrated lysate was filtrated through a 0.22-µm filter again before further purification. Then, phages were purified by CsCl density gradient ultracentrifugation (with three solutions in densities of 1.7,1.5,1.3 g/mL) at 24,000 rpm, 4°C, for 2.5 h and then dialyzed with molecular weight cut-off of 50-kDa dialysis tube with adjusted saline-magnesium (SM) buffer (100 mM NaCl, 8 mM MgSO_4_, and pH 7.5) to remove the residual CsCl three times. Purified phage was titrated, and the endotoxin level was detected by an endotoxin detection kit (Bioendo). The purified phage preparation shows high titer (2*10^11^ PFU/mL) and low endotoxin level (20 EU per 10^9^ PFU).

### Animals

Female ICR mice (6–8 weeks old; weigh 27–32 g) were purchased from Pengyue Experimental Animal Breeding, Co. (Jinan, Shangdong, China). The animals were housed under a 12-h dark-light cycle with free access to a standard diet and water. All animal experiments were approved by the Shandong Academy of Pharmaceutical Sciences Animal Ethics Committee.

### Safety study of phage PA_LZ7 *in vivo*

To evaluate the safety of phage PA_LZ7 in mice, 24 female ICR mice (6–8 weeks, weigh 27–32 g) were randomly divided into four groups, including vehicle control group (phosphate buffer saline [PBS] buffer: 20 mM Na_2_HPO_4_, 3.5 mM KH_2_PO_4_, 274 mM NaCl, 5.3 mM KCl, pH 7.5), 2*10^8^ (LD), 2*10^10^ (MD), and 2*10^11^ PFU/kg (HD). Mice were administered with PBS or phage through the tail vein at 10 mL/kg. Two doses of phage were given to the mice at the first day and the third day separately. Mice were sacrificed 24 h post second administration.

During the whole period of the experiment, the animals were observed daily to detect any signs of toxicity.

#### Clinical signs

The animals were observed continuously for any clinical signs or mortality for the first 6 h after the first administration; next, they were examined once daily following administration. The animals were observed for clinical signs, including changes in appearance, posture, movement, urine, body surface, and fluid secretion/excretion, throughout the experimental period.

#### Body mass

Animals were weighed before administration (D0) and on day 3.

#### Histopathologic analysis

Before necropsy, all surviving animals were fasted overnight (for 16–20 h) and anesthetized using intraperitoneal injections with Zoletil (Virbac, Carros, France)/xylazine for rats. After anesthesia was confirmed, exsanguination was performed to euthanize the animals. An initial inspection was then made of the body surface, subcutis, head, and all internal organs of the abdominal and thoracic cavities. Next, the brain, liver, lung, heart, spleen, kidneys, thymus, epididymis, ovaries, uterus, duodenum, ileum, jejunum, cecum, colon, and rectum were removed and examined separately. Then, the brain, liver, lung, heart, spleen, kidneys, and thymus were weighed.

Histological examination of organs was done as described previously (16). Briefly, tissues were fixed with 10% formalin and embedded in paraffin wax. Serial sections of 3-µm thickness were cut using a microtome, de-paraffinized, rehydrated, and stained with HE. For histological changes, the tissue sections were examined under the Nikon E100 microscope (Nikon, Tokyo, Japan).

#### Hematology and serum biochemistry

Blood samples were collected from the abdominal aorta of all mice scheduled for necropsy under deep anesthesia. Before the blood (about 0.3 mL) was collected into tubes, the tubes contained potassium EDTA as an anticoagulant for hematologic analysis using a hematology analyzer (XT-2000 iV, Sysmex Corporation, Kobe, Hyogo, Japan). About 0.5 mL of blood was collected from each animal into tubes containing sodium citrate and was analyzed using an automated CA1500 blood coagulation analyzer (Sysmex Corporation, Kobe, Hyogo, Japan). Approximately 0.8 mL of blood was collected from each animal into tubes for biochemical analysis, which employed an automated 7180 clinical chemistry analyzer (Hitachi, Tokyo, Japan).

### PK study of phage PA_LZ7 *in vivo*

To evaluate the PK of phage PA_LZ7 in mice, 36 female ICR mice (6–8 weeks, weigh 27–32 g) were randomly divided into four groups, including vehicle control, LD, MD, and HD groups. Blood was collected from the retro-orbital plexus at 3 min and 1, 6, and 24 h following the first intravenous administration. Three mice were sampled for each group at each time point, and all blood was anticoagulated with heparin. Samples at 1 and 24 h were also used for cytokine quantification. At 1, 24, or 72 h, mice were sacrificed (*n* = 3 per time point), and tissues (i.e., heart, lung, kidney, spleen, brain, and liver) were harvested and homogenized in SM buffer for phage PA_LZ7 quantification.

Samples were generally analyzed for phage enumeration with the double agar overlay method as follows: 20-µL plasma was transferred to a 1.5-mL Eppendorf tube, and 0.48-mL SM buffer was added; after vortexing, samples were passed through 0.22-µm filters; tissues were harvested and homogenized in SM buffer with a ratio of 1:5 (mass/volume); then, samples were passed through 0.22-µm filters; following a 10-fold serial dilution, 10 µL was added to their corresponding host bacterium agar overlays. The LLQ was 5,000 and 1,200 PFU/mL (i.e., 1 PFU per plate) for plasma and tissue samples, respectively.

For the PK study, regression analysis on the phage titer in plasma over time was performed using a one-phase decay model (16). The one-phase decay takes the form of Q_t_ = Q_0_*e ^*−kt*^, where Q*_t_* and *k* represent the relative phage titer at time *t* and the elimination rate constant (in h^−1^), respectively (Q_0_ denoted the phage titer at time = 0). The elimination half-life (T_1/2_) equals *ln*2/*k*. The regression was performed using Prism software version 7.04 for Windows (GraphPad Software, Inc., CA, USA).

### Cytokine quantification

The cytokine level of mice was evaluated by V-PLEX Proinflammatory Panel 1 Mouse Kit with measurements by Meso QuickPlex SQ120 following the manufacturer’s instructions (Meso Scale Discovery, Rockville, MD, USA).

### Data analysis

Comparisons were performed by one-way analysis of variance with Bonferroni's multiple comparison test and Student's *t*-test. All statistical analyses were performed using Prism 7.04 (GraphPad, San Diego, CA, USA), and differences with *P* < 0.05 were considered statistically significant.

## SUPPLEMENTAL MATERIAL

Fig. S1 spectrum.01882-23-s0001.pdf Lytic effect of phage PA_LZ7 against PAO1 *in vitro*.

ASM does not own the copyrights to Supplemental Material that may be linked to, or accessed through, an article. The authors have granted ASM a non-exclusive, world-wide license to publish the Supplemental Material files. Please contact the corresponding author directly for reuse.
