# Supplementary material for: Pharmacokinetics and safety evaluation of intravenously administered Pseudomonas phage PA_LZ7 in a mouse model
Source: Microbiol Spectr. 2023 Nov 28;12(1):e01882-23. doi: 10.1128/spectrum.01882-23 (PMC10783130; doi:10.1128/spectrum.01882-23)
Supplement: Fig. S1 — Lytic effect of phage PA_LZ7 against PAO1 in vitro. [file spectrum.01882-23-s0001.pdf]

Supplemental Material

Pharmacokinetics and safety evaluation of intravenously administered *Pseudomonas* phage PA_LZ7 in a mouse model

Siyun Wang[1], Xin Tan[2*], Ziqiang Liu[2], Hui Ma[3,4], Tianbin Liu[3,4], Yongqing Yang[2], Yong Ying[3,4], Ruyue Gao[2], Daizhou Zhang[3,4], Yingfei Ma[2], Kai Chen[3,4], Lin Lin[3,4*], Zhihuan Jiang[3,4*], Jialin Yu[1*]

1 Department of Neonatology, Children's Hospital of Chongqing Medical University, National Clinical Research Center for Child Health and Disorders, Ministry of Education Key Laboratory of Child Development and Disorders, Chongqing Key Laboratory of Child Infection and Immunity, Chongqing Key Laboratory of Pediatrics, Chongqing, China.

2 Shenzhen Key Laboratory of Synthetic Genomics, Guangdong Provincial Key Laboratory of Synthetic Genomics, CAS Key Laboratory of Quantitative Engineering Biology, Shenzhen Institute of Synthetic Biology, Shenzhen Institutes of Advanced Technology, Chinese Academy of Sciences, Shenzhen, China

3 New Drug Evaluation Center of Shandong Academy of Pharmaceutical Sciences, Shandong Academy of Pharmaceutical Sciences, Ji'nan, China

4 Shandong Innovation Center of Engineered Bacteriophage Therapeutics, Ji'nan, China

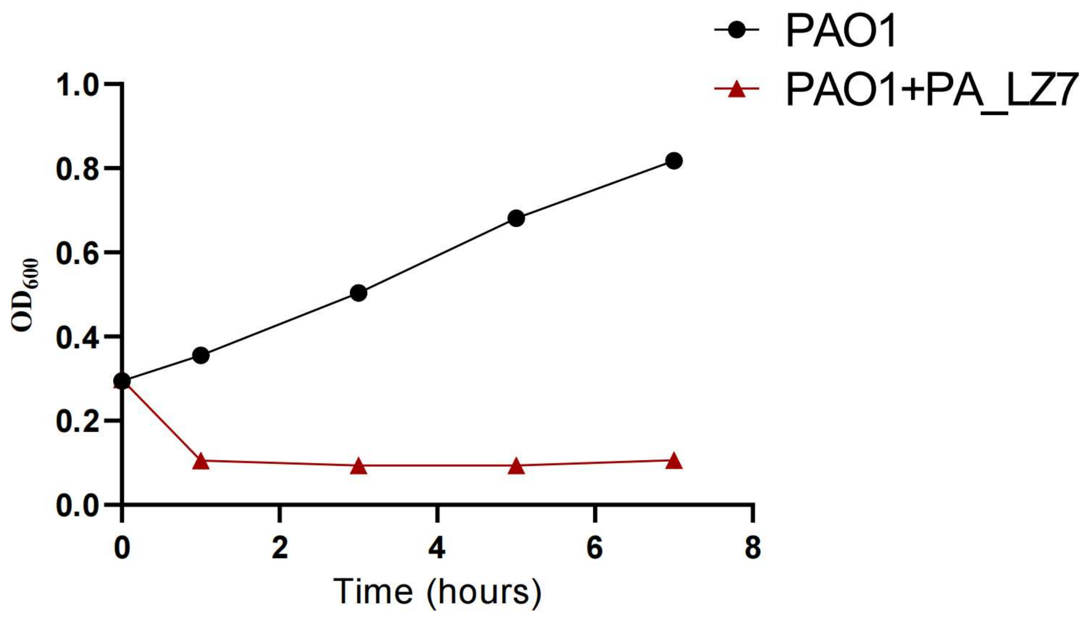

Figure S1. Lytic effect of phage PA_LZ7 against PAO1 *in vitro*. Growth kinetics of planktonic PAO1 in presence of phage PA_LZ7. Exponentially growing PAO1 (OD600=0.3) were infected by phage PA_LZ7 at an MOI of 1:100.
